# Axillary lymph nodes on PET in Hodgkin lymphoma after COVID‐19 vaccination

**DOI:** 10.1002/jha2.297

**Published:** 2021-10-13

**Authors:** Anne I. J. Arens, Konnie M. Hebeda, Martin Hutchings, Wouter J. Plattel, Wendy B. C. Stevens

**Affiliations:** ^1^ Department of Radiology and Nuclear Medicine Radboud University Medical Center Nijmegen The Netherlands; ^2^ Department of Pathology Radboud University Medical Center Nijmegen The Netherlands; ^3^ Department of Haematology Rigshospitalet Copenhagen Denmark; ^4^ Department of Haematology University Medical Center Groningen University of Groningen Groningen The Netherlands; ^5^ Department of Haematology Radboud University Medical Center Nijmegen The Netherlands


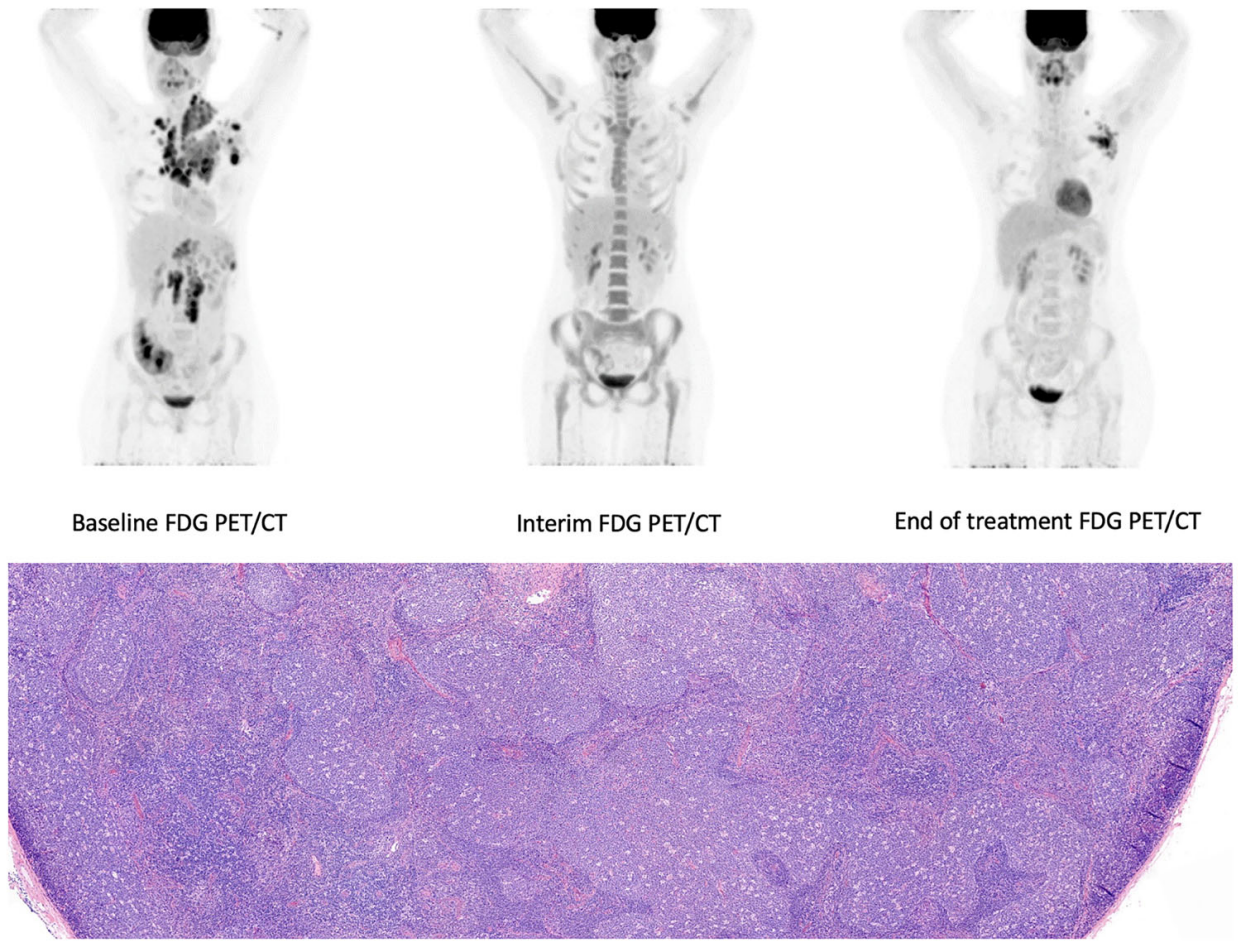


A 21‐year‐old woman presented with nodular sclerosis Hodgkin lymphoma stage IIIA, IPS 2 with a baseline ^18^F‐fluorodeoxyglucose positron emission tomography and computed tomography (FDG PET/CT) showing initial involved lymph nodes in both sides of the neck, left axilla, mediastinum, hili, para‐aortic, left iliac, and involvement of the spleen. After one course of BrAVD (brentuximab‐vedotin, adriamycine, vinblastine, dacarbazine), the interim FDG PET/CT scan was negative, classified as a complete metabolic response. A third FDG PET/CT scan was performed after completion of her chemotherapy consisting of six courses of BrAVD. This scan showed a reappearance of FDG avid lymph nodes, with a Deauville score of 5, in the original sites in the left axilla and neck. The most FDG avid lymph node was excised and revealed florid follicular hyperplasia instead of recurrence of Hodgkin lymphoma.

In hindsight, she received a COVID‐19 vaccination in her left arm several weeks prior to the last FDG PET/CT scan. In the meantime, various articles were published that relate the occurrence of FDG avid ipsilateral axillary and neck lymph nodes to COVID‐19 vaccination of several manufacturers. We, therefore, concluded all FDG avid lymph nodes to be triggered by the vaccination. FDG PET/CT scan is valuable in steering therapy in Hodgkin lymphoma patients. However, enhanced FDG uptake only indicates that enhanced glucose metabolism and false positive results are possible, necessitating good clinical correlation. Documentation of the vaccination status including date(s), side, and manufacturer is of importance. In cancer patients, the side and timing of the vaccination should be discussed with the patient to prevent concern of recurrence and avoid unnecessary diagnostics and as in our case.

